# Medicare Savings Programs, Part D Low-Income Subsidy, and Characteristics of Medicare Beneficiaries

**DOI:** 10.1093/geroni/igaf122.3623

**Published:** 2025-12-31

**Authors:** Nicha Thiamwong, Boon Peng Ng

**Affiliations:** University of Central Florida, Orlando, Florida, United States; University of Central Florida, Orladno, Florida, United States

## Abstract

Medicare Savings Programs and Part D Low-Income Subsidy program assist low-income beneficiaries with healthcare and prescription costs. However, information about eligible Medicare beneficiaries’ characteristics is limited. This study aimed to examine the socio-demographic characteristics of beneficiaries in these programs. The 2022 Medicare Current Beneficiary Survey Public Use File, a nationally representative data, of Medicare beneficiaries aged ≥65 years (n = 10,186), was used. A three-level categorical dependent variable was created: (1) has Medicare Savings Program and Part D Low-Income Subsidy, (2) has either program, and (3) has neither (reference group). A survey-weighted multinomial regression model was used to examine relationships between socio-demographics and the dependent variable, adjusted for health conditions. Of the study beneficiaries, 12.0% had both programs, 2.1% had either, and 85.9% had neither. About 25% of those with incomes < =100% of the Federal Poverty Level (FPL) did not enroll. Non-Hispanic Blacks (NHB), Hispanics, and Other were more likely than non-Hispanic Whites to have both programs (e.g., for NHB, OR = 2.74, 95% CI [2.01-3.64]). Beneficiaries with less than a high school education were more likely to have both (OR = 2.14, [1.61-2.84]) or have either program (OR = 2.37, [1.33-4.24]) than those with higher education. Beneficiaries with income >200% of the FPL were less likely to have both (OR = 0.01, [0.01-0.02]) or either program (OR = 0.07, [0.04-0.13]) than those < =100% of the FPL. Both programs aided ∼15% (representing 7.5 million) of beneficiaries, mainly from lower socio-demographic groups. However, about 1 million beneficiaries had either program, and some eligible individuals were unenrolled, emphasizing policy reforms to improve enrollment processes.

